# Population structure of eleven Spanish ovine breeds and detection of selective sweeps with BayeScan and hapFLK

**DOI:** 10.1038/srep27296

**Published:** 2016-06-07

**Authors:** A. Manunza, T. F. Cardoso, A. Noce, A. Martínez, A. Pons, L. A. Bermejo, V. Landi, A. Sànchez, J. Jordana, J. V. Delgado, S. Adán, J. Capote, O. Vidal, E. Ugarte, J. J. Arranz, J. H. Calvo, J. Casellas, M. Amills

**Affiliations:** 1 Department of Animal Genetics, Center for Research in Agricultural Genomics (CSIC-IRTA-UAB-UB), Campus Universitat Autònoma de Barcelona, Bellaterra 08193, Spain; 2 CAPES Foundation, Ministry of Education of Brazil, Brasilia D. F., Zip Code 70.040-020, Brazil; 3 Departamento de Genética, Universidad de Córdoba, Córdoba 14071, Spain; 4 Unitat de Races Autòctones, Servei de Millora Agrària i Pesquera (SEMILLA), Son Ferriol 07198, Spain; 5 Departamento de Ingeniería, Producción y Economía Agrarias, Universidad de La Laguna, 38071 La Laguna, Tenerife, Spain; 6 Departament de Ciència Animal i dels Aliments, Universitat Autònoma de Barcelona, Bellaterra 08193, Spain; 7 Federación de Razas Autóctonas de Galicia (BOAGA), Pazo de Fontefiz, 32152 Coles. Ourense, Spain; 8 Instituto Canario de Investigaciones Agrarias, La Laguna 38108, Tenerife, Spain; 9 Departament de Biologia, Universitat de Girona, Girona 17071, Spain; 10 Neiker-Tecnalia, Campus Agroalimentario de Arkaute, apdo 46 E-01080 Vitoria-Gazteiz (Araba), Spain; 11 Departamento de Producción Animal, Universidad de León, León 24071, Spain; 12 Centro de Investigación y Tecnología Agroalimentaria de Aragón (CITA), Unidad de Tecnología en Producción Animal, Avda. Montañana, 930, 50059 Zaragoza, Spain

## Abstract

The goals of the current work were to analyse the population structure of 11 Spanish ovine breeds and to detect genomic regions that may have been targeted by selection. A total of 141 individuals were genotyped with the Infinium 50 K Ovine SNP BeadChip (Illumina). We combined this dataset with Spanish ovine data previously reported by the International Sheep Genomics Consortium (N = 229). Multidimensional scaling and Admixture analyses revealed that Canaria de Pelo and, to a lesser extent, Roja Mallorquina, Latxa and Churra are clearly differentiated populations, while the remaining seven breeds (Ojalada, Castellana, Gallega, Xisqueta, Ripollesa, Rasa Aragonesa and Segureña) share a similar genetic background. Performance of a genome scan with BayeScan and hapFLK allowed us identifying three genomic regions that are consistently detected with both methods *i.e.* Oar3 (150–154 Mb), Oar6 (4–49 Mb) and Oar13 (68–74 Mb). Neighbor-joining trees based on polymorphisms mapping to these three selective sweeps did not show a clustering of breeds according to their predominant productive specialization (except the local tree based on Oar13 SNPs). Such cryptic signatures of selection have been also found in the bovine genome, posing a considerable challenge to understand the biological consequences of artificial selection.

Since their domestication in the Fertile Crescent, sheep have been bred for producing milk, meat and wool^1^. Artificial selection for these and other phenotypic traits probably began thousands of years ago by keeping as breeders individuals with certain external features (*e.g.* color, size, morphology etc) and productive abilities (rapid growth and high fertility). The speed of this process of genetic change accelerated enormously in the last decades as a consequence of the implantation of intensive breeding schemes based on artificial insemination, extensive trait and genealogical recording, and the introduction of best linear unbiased predictor approaches to estimate genetic values^2^. Certain cosmopolitan breeds became strongly specialized in either meat, wool or dairy production, while others, with a more local distribution, kept a more balanced production profile. Currently, in Spain there are 43 officially recognized ovine breeds that encompass 16 million individuals (the 2^nd^ largest census of the European Union) and produce around 23,000, 120,000 and 550,000 metric tonnes of wool, meat and milk, respectively (FAOSTAT, http:// faostat.fao.org).

Given the quantitative nature of production traits, it can be anticipated that most of the genetic changes introduced by artificial selection in the genomes of meat and dairy sheep are driven by polygenic adaptation *i.e.* shifts in the allele frequencies of hundreds or thousands of loci that have small effects on the selected trait^3^. In some instances, however, selection may act on a new single variant that has a major effect on a phenotype of interest^4^. In this particular scenario, a hard selective sweep takes place, leaving one or several genetic signatures (*i.e.* an excess of rare polymorphisms or derived alleles, high genetic differentiation, extended linkage disequilibrium, etc) that can be recognized with appropriate statistical methods^5^. In sheep, several genome scans aimed to identify selection signatures related with fat deposition^6^, morphology and color^7^, dairy production^7^,^8^, presence of horns^2^ and adaptation to climate conditions^9^ have been carried out so far, leading to the identification of a diverse array of selective sweeps scattered throughout the ovine genome. The aim of the current work was to analyse the population structure of eleven Spanish sheep breeds and to identify selection signatures produced by artificial selection for growth and milk traits.

## Results and Discussion

### Analysis of the population structure of eleven Spanish ovine breeds

**Figure 1:**
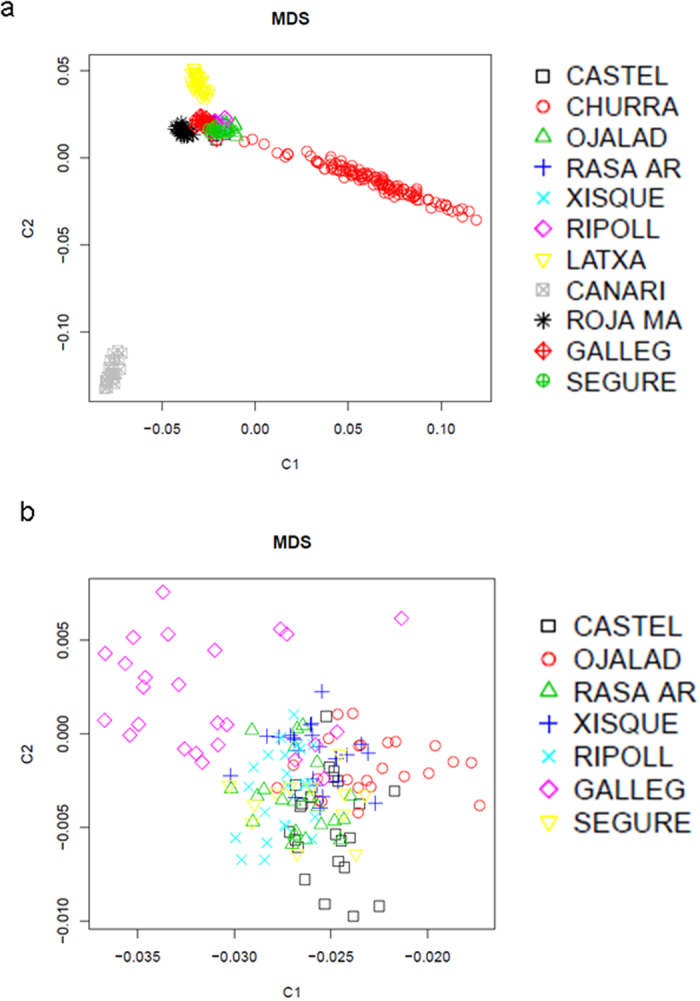
(**a**) Multidimensional scaling plot based on genome-wide identity-by-state pairwise distances inferred with PLINK. This graph displays the genetic relationships between Castellana (CASTEL), Churra (CHURRA), Ojalada (OJALAD), Rasa Aragonesa (RASA AR), Xisqueta (XISQUE), Ripollesa (RIPOLL), Latxa (LATXA), Canaria de Pelo (CANARI), Roja Mallorquina (ROJA MAR), Gallega (GALLEG) and Segureña (SEGURE) sheep. (**b**) The same multidimensional scaling plot shown in Fig. 1a, but excluding Churra, Latxa, Canaria de Pelo and Roja Mallorquina breeds.

**Figure 2:**
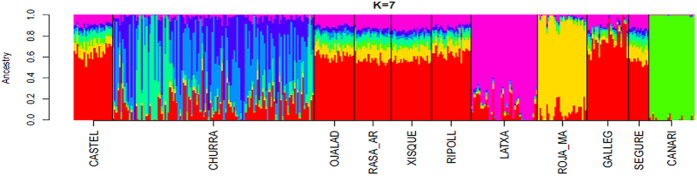
Admixture analysis of 11 Spanish ovine breeds: Castellana (CASTEL), Churra (CHURRA), Ojalada (OJALAD), Rasa Aragonesa (RASA AR), Xisqueta (XISQUE), Ripollesa (RIPOLL), Latxa (LATXA), Canaria de Pelo (CANARI), Roja Mallorquina (ROJA MAR), Gallega (GALLEG) and Segureña (SEGURE). We set the number of clusters to K = 7 (this K-value had the lowest cross-validation error).

The multidimensional scaling (MDS, Fig. 1) plot analysis of 11 Spanish sheep breeds with a wide geographic distribution (Supplementary Fig. S1) revealed that the Canaria de Pelo breed is highly differentiated from the remaining populations. We also observed a scattered and divergent cluster represented by the Churra breed. The Roja Mallorquina and Latxa breeds also showed a significant genetic differentiation, while the remaining seven breeds were mixed in a single cluster and they could not be easily distinguished from each other (Fig. 1a). When making a second MDS analysis of the aforementioned seven breeds (Fig. 1b), we were able to distinguish the Gallega sheep from the other populations. These results were consistent with the Admixture analysis (Fig. 2), which showed that Canaria de Pelo, Roja Mallorquina, Latxa and Churra breeds have a well defined genetic identity. In contrast, Castellana, Ojalada, Rasa Aragonesa, Xisqueta, Ripollesa, Gallega and Segureña sheep share a similar genetic background. These findings are consistent with the weak population structure observed in ovine breeds with a worldwide distribution^10^.

There are reports that indicate that Canaria de Pelo, the only hair sheep breed in Spain, became extinct in the Canary islands during the 16–17^th^ centuries and that current populations descend from Pelibuey sheep brought from Venezuela^11^. This Pelibuey sheep, in turn, may have a Canarian origin because this Atlantic archipelago was an obliged port-of-call for the ships in route to the New World during the 15^th^ century and onwards^11^. Hair sheep are the most widespread race in Africa because of their excellent adaptation to the highly humid tropical forest^12^. Linguistic and genetic evidences connect the aborigin Canarian population with the Imazighen peoples indigenous to North Africa^13^. In consequence, we attribute the high genetic differentiation of the Canaria de Pelo sheep to the fact that it has an African rather than Iberian origin. Geographic isolation, until the discovery of the Canarian archipelago by the Spanish in the 15^th^ century, combined with the occurrence of population bottlenecks may have also contributed to enhance genetic divergence^14^,^15^.

Roja Mallorquina, Churra and Latxa sheep also had a defined genetic identity (Figs 1 and 2). Roja Mallorquina sheep display phenotypic features that are distinctive of certain breeds from North Africa and Asia such as a fat triangular tail and a red color. Indeed, fat-tailed sheep are particularly abundant in Lybia, Tunisia and Algeria and it is assumed that they were introduced from the Middle East^12^. Churra is one of the most important milking sheep breeds in Spain and it is mainly raised in Castile and Leon, while Latxa has a lower census and a more restricted geographic distribution in Navarra and the Basque Country. The classical phenotypic classification of ovine Spanish breeds proposed by Antonio Sánchez-Belda highlights the existence of four main lineages^16^: (1) Churro (Churra and Latxa breeds, that have a coarse wool), (2) Merino (not represented in our dataset), (3) Medium Fine Wool (Segureña, Gallega, Ripollesa, Rasa Aragonesa, Castellana and others), and (4) Iberian (Xisqueta, Ojalada and others). Our genetic data do not support the existence of a substantial genetic divergence between the Medium Fine Wool and the Iberian breeds. As shown in Fig. 1b, Ojalada and Xisqueta sheep are not significantly differentiated from their Segureña, Ripollesa, Gallega, Rasa Aragonesa and Castellana counterparts, suggesting that these populations belong to a single genetic lineage. It can be observed, however, that the Gallega breed shows a certain level of genetic divergence when compared to the remaining six breeds (Fig. 1b). Moreover, our data do not show a common clustering of the the Latxa and Churra sheep (Figs 1 and 2), suggesting that they do not have a common origin.

### Detection of selective sweeps with BayeScan and hapFLK

**Figure 3:**
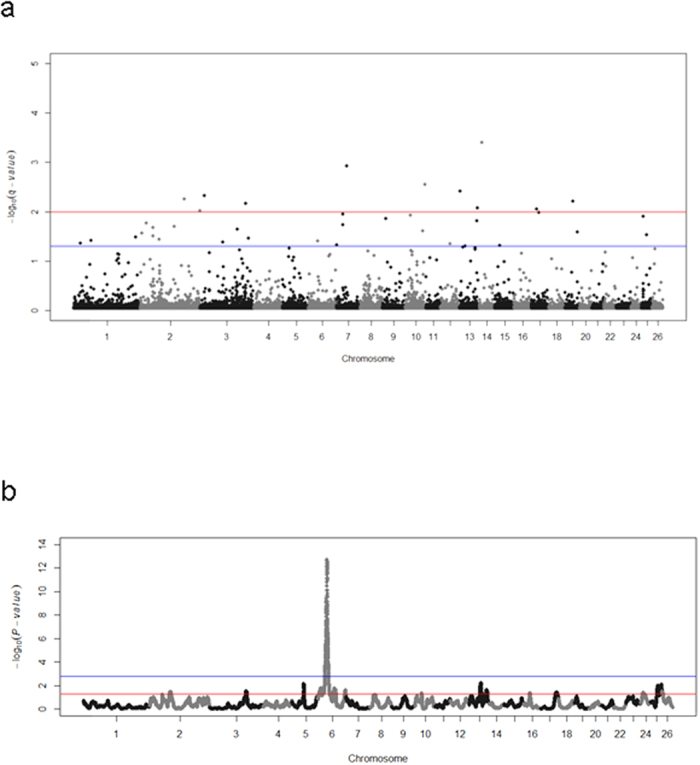
Whole-genome scan for selective sweeps. The two analyses were based on either the F_ST_-outlier method implemented in BayeScan (**a**) or the hapFLK statistic (**b**). In the BayeScan analysis, the red and blue lines indicate the thresholds of significance set at 0.05 and 0.01 after correction for multiple testing (q-values), respectively. In the hapFLK analysis, the red and blue lines indicate the thresholds of significance set at 0.05 before (nominal P-value) and after (q-value) correction for multiple testing, respectively. Genomic coordinates and statistical significance (−log_10_ *P*-values) are plotted in the x- and y-axis, respectively.

Our study was designed to identify selective sweeps in dairy and non-dairy sheep breeds with a similar genetic background (all of them were Spanish) in order to minimize the confounding effects of ascertainment bias^17^. Canaria de Pelo was excluded from selection analyses because of its high genetic divergence with regard to the remaining Spanish breeds (Fig. 1). With BayeScan, we detected 39 genomic regions distributed in 15 chromosomes that displayed significant evidences of being under selection (Table 1, Fig. 3a). The sign of α was always positive indicating that, in all cases, we had detected the effects of directional selection. Comparison of our results with those reported in a set of dairy and non-dairy ovine breeds with diverse origins^8^ showed some matches on Oar2, Oar3, Oar6 and Oar15 (Table 1). We also compared our data with results generated in a worldwide sample of breeds differing at many phenotypes^7^. Interestingly, we found positional coincidences amongst putative selective sweeps detected on Oar2, Oar3, Oar6, Oar10, Oar14 and Oar19 (Table 1). Co-localizations between selective sweeps identified in different studies should not be taken as irrefutable proof of their existence, because they may emerge just by chance (though the probability of the occurrence of such random co-localizations might be low). In the current work, performance of a circular permutation test (see Methods) demonstrated that the number of positional coincidences detected by us exceeds what would be expected by chance (bootstrapped *P*-value < 0.05).

The hapFLK analysis (Table 2, Fig. 3b) yielded fewer positive results than analyses based on BayeScan (Table 1) or FLK (Supplementary Table S1). Consistent with this, in a previous genome scan focused on layer chicken populations^18^, the numbers of SNPs under selection detected with Bayescan (212,765 SNPs) and FLK (155,712 SNPs) were 7-fold and 5-fold larger than those detected with hapFLK (28,557 SNPs). The cause of these discrepancies might be that hapFLK is less sensitive than BayeScan to distortions caused by demography (*e.g.* bottlenecks, differences in effective population sizes amongst breeds, etc) and hierarchical population structure^2^,^7^. Moreover, hapFLK is also expected to be more stringent than FLK because it implements a multipoint linkage disequilibrium model^19^ that takes into account the haplotype structure of the sample. Indeed, our hapFLK analysis only yielded one significant selective sweep at Oar6 (4.3–49.9 Mb) after correction for multiple testing. A coincident selective sweep in the 37–38 Mb interval of Oar6 was previously found by analysing a set of Alpine ovine breeds with the hapFLK statistic^2^.

As shown in Table 2, there was a substantial positional concordance between the set of selective sweeps detected with hapFLK and those described in previous reports^7^,^8^. This level of coincidence also exceeded what would be expected just by chance (bootstrapped *P*-value < 0.05) based on a circular permutation test. The set of Spanish populations analysed in our study is considerably different to those employed by other authors *i.e.*10 European breeds (2 of them with a Spanish origin)^8^, and 29 international breeds (only 1 Spanish breed)^7^. Although drift and migration can generate local signatures that can be confounded with those produced by selection, in principle we do not expect distantly related sets of populations to share such demographic signals. Moreover, the coincident F_ST_-outlier signals found by us and others^2^,^7^,^8^ suggest that at least part of the selective sweeps detected with BayeScan are true positives (despite the fact that they were not detected with hapFLK).

### Three selective sweeps are consistently detected with BayeScan and hapFLK

**Figure 4:**
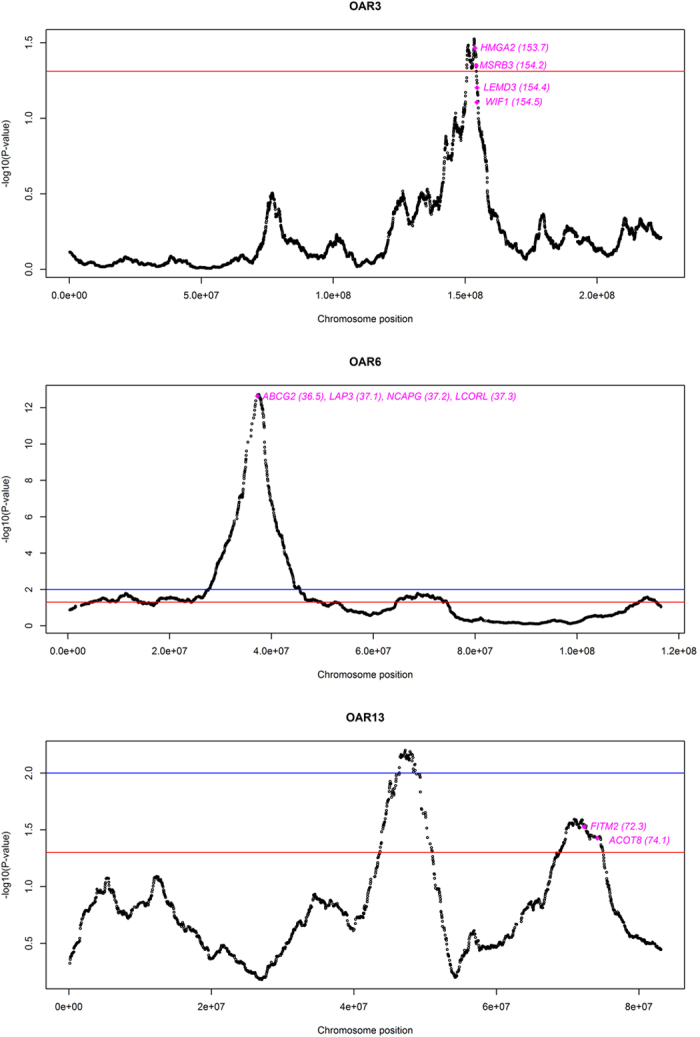
A detailed view of the putative selective sweeps on Oar3, Oar6 and Oar13 detected with the HapFLK statistic and confirmed with BayeScan. The red and blue lines indicate the thresholds of significance set at 0.05 before (nominal P-value) and after (q-value) correction for multiple testing, respectively. Genomic coordinates and statistical significance (−log_10_ *P*-values) are plotted in the x- and y-axis, respectively. The approximate location of the candidate genes discussed in the current work is indicated (in Mb).

When we considered the BayeScan data set and the selective sweeps detected with hapFLK that are significant at the nominal level (Table 2, Fig. 4), positional coincidences were identified on Oar3 (150–154 Mb), Oar6 (4.3–49.9 Mb), and Oar13 (68.8–74.9 Mb). Next, we will examine more thoroughly a set of physiological and positional candidate genes whose patterns of variation could have been potentially modified by selection.

#### Selective sweep on Oar3

The Oar3 (150–154 Mb) region co-localizes with a pleiotropic bovine quantitative trait locus (QTL) for birth weight, calving ease direct, marbling and ribeye muscle area^20^ as well as with a second bovine QTL for fat yield^21^. There are several genes that may explain the existence of a selective sweep in this genomic region. The high mobility group AT-hook 2 (*HMGA2*, 153.7 Mb) gene is particularly relevant because polymorphism at this transcriptional regulator has been associated with height in humans and body size in horses and dogs^20^. Moreover, the inactivation of *HMGA2* in mouse leads to the pygmy phenotype, characterized by a substantial decrease in body size and adiposity and defective spermatogenesis^22^,^23^. Another gene of interest is the WNT Inhibitory Factor 1 (*WIF1*, 154.5 Mb) locus, that encodes a molecule inhibiting extracellular WNT signaling, and that has been identified as positively selected in domestic cattle^24^. Interestingly, the WNT effector pathway is essential for the initiation of embryonic mammary organogenesis and the maintenance of stem cells, and it may also regulate post-natal ductal and alveolar development^25^. Finally, it is worth to mention the methionine sulfoxide reductase B3 (*MSRB3*, 154.2 Mb) and the LEM domain containing 3 (*LEMD3*, 154.4 Mb) loci, that are involved in cell growth^26^ and skeletal development^27^, respectively.

#### *Selective sweep on Oar6*

The Oar6 selective sweep contains several genes that may have been affected by selection *i.e.* the non-SMC condensin I complex, subunit G (*NCAPG*, 37.2 Mb), the ligand dependent nuclear receptor corepressor-like (*LCORL*, 37.3 Mb), the leucine aminopeptidase 3 (*LAP3*, 37.1 Mb) and the ATP-binding cassette, sub-family G (WHITE), member 2 (*ABCG2*, 36.5 Mb) loci. Indeed, the *NCAPG/LCORL* gene pair has been reported as a selection target in many genome scans. *LCORL* is a co-repressor of ligand-regulatable transcriptional factors, such as the estrogen α and thyroid hormone receptors, and plays a fundamental role in hepatic lipogenesis^28^. More importantly, variation at *LCORL* has been associated with height in humans^29^ and horses^30^, and with vertebrae number in pigs^31^. Similarly, *NCAPG* plays a key role in mitotic cell division and affects post-natal growth^32^. Other genes of interest are *ABCG2*, a molecule transporter that has been associated with milk yield and composition^33^, and *LAP3.* This latter gene displays a selection signature in Holstein cattle and its variability is associated with diverse milk traits^24^. Interestingly, the bovine chromosome 6 region containing *LCORL*, *NCAPG, LAP3* and *ABCG2* overlaps with several quantitative trait loci for growth, carcass quality, feed efficiency, reproduction and milk traits^34^,^35^,^36^,^37^.

At this point, is difficult to know if selection on Oar6 is targeting one or several loci. In principle, we would favour this second scenario because data generated by us and others evidence that the size of the Oar6 region under selection is considerably large suggesting that it may have been produced by the superposition of several overlapping peaks (Fig. 4). The multiple associations with production traits observed in cattle would also favour this hypothesis, although we cannot rule out the possibility of selection acting on a single gene with pleiotropic effects.

#### Selective sweep on Oar13

Within the Oar13 selective sweep (68–74 Mb), there are two genes related with lipid metabolism *i.e*. the fat storage-inducing transmembrane protein 2 (*FITM2*, 72.3 Mb) and the acyl-CoA thioesterase 8 (*ACOT8*, 74.1 Mb) loci. The *FITM2* protein is located in the endoplasmic reticulum and induces the packaging of triglycerides as lipid droplets^38^. This mechanism could be of importance in the mammary gland, since lipids are secreted as droplets that bud from the epithelial cells. The *ACOT8* molecule hydrolyzes medium- to long-chain acyl-CoAs and its overexpression has been shown to abolish peroxisomal fatty acid β-oxidation and enhance lipid accumulation in droplets^39^. Thus, these two loci may have effects on milk lipid content. Though Spanish sheep have not been specifically selected for milk fat content, the negative and moderate correlation of this trait with milk yield offers a possible explanation for our findings.

### Relationship between variation at markers mapping to putative selective sweeps and productive specialization

The main goal of our study was to map selective sweeps related with the genetic improvement of Spanish sheep for milk traits. Latxa and Churra sheep produce around 180 kg (in 140 days) and 117 kg (in 120 days) of milk (Spanish Ministry of Agriculture, Food and Environment web, http://www.magrama.gob.es), respectively. Certainly, these numbers are significantly lower than milk yield registers of cosmopolitan highly specialized breeds (*e.g.* Lacaune sheep produce 350 kg milk in 150 days). However, in the last two decades the milk production of Spanish dairy sheep breeds has been the subject of intensive breeding programs. For instance, the Churra breed has experienced a 15–20% increase in milk production during the last 25 years (Churra Breeding Association web, http://www.anche.org).

In the light of these facts, we expected to find selective sweeps related with meat *vs* milk production in our dataset. When we built a population tree based on SNPs mapping to the three selective sweeps, we did not observe a clustering of the Churra and Latxa dairy breeds, though they were located in close positions (Supplementary Fig. S2). Consistently, local trees based on SNPs that mapped to the Oar3 and Oar6 selective sweeps did not show a clustering of Churra and Latxa. In contrast, both breeds grouped together in the local tree based on SNPs located within the Oar13 selective sweep. Moreover, the analysis of the allele frequencies of SNPs mapping to the Oar3, Oar6 and Oar13 selective sweeps did not reveal any meaningful pattern (Supplementary Fig. S3). These inconclusive results could be due to the limited power and the stringency of our experiment. We may have missed many selective sweeps that did not reach statistical significance due to the moderate sample size employed in our study or because they were not simultaneously identified with BayeScan and hapFLK. Genetic heterogeneity amongst breeds, where distinct mutations have similar effects on milk yield or growth, could be another reason. It is also possible that the selective sweeps we have detected do not have any relationship with meat or milk production but with other traits (*e.g.* morphology, adaptation, reproduction, disease resistance) that we did not take into consideration in our selection analysis. A fourth factor could be that artificial selection for meat and dairy traits has mainly evolved through polygenic adaptation, shifting the allele frequencies of hundreds or thousands of loci instead of fixing novel mutations with major phenotypic effects. Finally, the methods used by us are good at detecting ongoing or recently completed selective sweeps but they have difficulties in identifying ancient sweeps that ended a long time ago^40^.

Though we have found patterns of variation on Oar3, Oar6, and Oar13 that are compatible with the occurrence of selective sweeps, it is difficult to envisage which set of phenotypes were really targeted by selection. Indeed, intensive selection of Spanish sheep breeds, as Churra and Latxa, for milk production is relatively recent (it began 2–3 decades ago) and genetic exchanges between dairy and non-dairy populations may have taken place, thus obscuring the effects of selection. Importantly, several of the selective sweeps detected with BayeScan and hapFLK contained genes encoding transcriptional regulators with effects on body size (*e.g. HGMA2* on Oar3 and *LCORL* and *NCAPG* on Oar6). This phenotype experienced a substantial reduction during the early times of domestication and subsequently increased as a consequence of artificial selection for growth rate. Changes in the selection pressure conferring a higher biological efficacy to a mutation that was previously deleterious are expected to generate hard sweep signatures^41^. Our finding, however, is difficult to interpret because the set of breeds employed in the current work do not differ substantially in terms of body size, weight or stature. Such cryptic selective sweeps have been also observed in cattle^41^, and so far their biological significance remains unknown. Noteworthy, neutral loci with low recombination rates may exhibit many of the features of positively selected genes, generating spurious signals in selective sweep scans. Given the intrinsic difficulties of interpreting selection mapping data, additional tools, such as genome-wide association studies based on high throughput genotyping or whole-genome sequencing data obtained from large reference populations, will be indispensable to uncover the biological meaning of selective sweep signatures.

## Materials and Methods

### Ethics statement

Blood samples were collected from sheep by trained veterinarians in the context of sanitation campaigns and parentage controls not directly related with our research project. In all instances, veterinarians followed standard procedures and relevant Spanish national guidelines to ensure an appropriate animal care.

### Nucleic acid purification and genotyping with the Ovine 50 K SNP BeadChip

Blood was extracted with Vacutainer tubes from 141 sheep corresponding to the Segureña (N = 12), Xisqueta (N = 25), Ripollesa (N = 23), Gallega (N = 25), Canaria de Pelo (N = 27), and Roja Mallorquina (N = 29) breeds. Leukocytes were purified from whole blood by carrying out several washing steps with TE buffer (Tris 10 mM, EDTA 1 mM, pH 8.0). In this way, a volume of TE was added to 500 μl blood and this mixture was vortexed and centrifuged at 13,000 rpm for 30 seconds. This procedure was repeated until a clean white pellet was obtained. Next, the cell pellet was resuspended in 200 μl cell lysis buffer (50 mM KCl, 10 mM Tris, 0.5% Tween 20) with 10 μl proteinase K (10 mg/ml) and incubated for 4 hours at 56 °C. One volume of phenol:chloroform:isoamyl alcohol (25:24:1) was added to the lysate, and the resulting mixture was vortexed and centrifuged at 13,000 rpm for 15 min. Subsequently, the aqueous upper layer was transferred to a fresh tube and 2 M NaCl (0.1 volumes) and absolute ethanol (2 volumes at -20 °C) were added. After a centrifugation step at 13,000 rpm for 30 min., the supernatant was discarded and salt contamination was removed by performing a washing step with 500 μl 70% ethanol. Finally, the DNA pellet was air-dried at room temperature, and resuspended in 50 μl milli-Q water.

Genomic DNA samples obtained in this way were typed for 54,241 SNPs with the Ovine 50 K SNP BeadChip following standard protocols (http://www.illumina.com). Moderate sample size and the low density of this genotyping platform may have limited to some extent the power of our experiment. However, this was the only high throughput SNP typing tool available at the time we initiated genotyping tasks. The GenomeStudio software (Illumina) was used to generate standard ped and map files as well as to perform sample and marker-based quality control measures (we considered a GenCall score cutoff of 0.15 and an average sample call rate of 99%). Genotyping data generated in the current work were submitted to the International Sheep Genomics Consortium database (ISGC, http://www.sheephapmap.org) and they should be available upon request.

### Population structure analyses

Besides the 50 K data generated in our project for six ovine breeds from Spain, in the population structure and selection analyses we also used existing 50 K data from 229 sheep belonging to the Ojalada (N = 24), Castellana (N = 23), Rasa Aragonesa (N = 22), Churra (N = 120) and Latxa (N = 40) breeds, that were kindly provided by the International Sheep Genomics Consortium. The Latxa and Churra sheep employed in the current work are specialized in milk production, whilst the remaining breeds form a heterogeneous group fundamentally devoted to the production of meat (non-dairy sheep). Noteworthy, the breeding schemes of the Segureña and Rasa Aragonesa are well established and mostly focused on growth and prolificacy traits, respectively. In contrast, those of the other six non-dairy breeds have a less advanced status.

Polymorphism 50 K data provided by the ISGC had been already filtered^10^. Taking into account that we could not replicate the same filtering criteria used by the ISGC (we did not have trios or a parallel typing platform to check genotype assignment consistency), we homogenized our (54,241 SNPs) and ISGC (49,304 SNPs) datasets by joining them with the PLINK V 1.07^42^ command ‒merge. This common datafile was subsequently filtered applying the following criteria. (1) All unmapped SNPs or those mapping to sexual chromosomes were removed; (2) SNPs with a genotyping rate lower than 90% or that failed the frequency test (setting a Minor Allele Frequency threshold of 0.05) were pruned; and (3) We also eliminated SNPs that did not pass the HWE test (*P* ≤ 0.001) because it is reasonable to assume that the main cause of HWE departures are genotyping errors^6^. After these filtering steps, a total of 43,343 SNPs were available for population structure and selection analyses. The sheep genome assembly v3.1 was used as a reference. The PLINK v1.07 program was used to perform a MDS analysis based on a matrix of genome-wide pairwise identity-by-state distances^42^. Besides, we carried out a clustering analysis with Admixture v1.23, which calculates maximum likelihood estimates of individual ancestries based on data provided by multiple loci^43^,^44^.

### Performance of a genome scan for selective sweeps

#### Identification of selective sweeps with BayeScan

Selection signatures were detected by using the F_ST_-outlier approach implemented in the BayeScan software^45^. This statistical methodology allows to identify loci that are under selection because they show F_ST_ coefficients that are significantly more different than expected under neutrality and a given demographic model. In this sense, genes under balancing or purifying selection are assumed to display too even allele frequencies across populations (low F_ST_), whilst those under local directional selection are expected to generate strong genetic differences (high F_ST_) between populations. With BayeScan^45^, F_ST_ coefficients are partitioned into a population-specific component (β), common to all loci, and a locus-specific component (α) shared by all the populations using a logistic regression. Allele frequencies are assumed to follow a Dirichlet distribution. Selection is detected when α is significantly different from zero *i.e.* the locus-specific component is necessary to explain the observed pattern of diversity. When α > 0 it is assumed that directional selection if acting on the locus under analysis, while α < 0 suggests balancing or purifying selection.

Standard PLINK files were converted to the BayeScan format with the PGDSpider v 2.0.7.3 software^46^. BayeScan analyses comprised 20 pilot runs of 5,000 iterations, a burn-in of 50,000 iterations, a thinning interval of 10 (5,000 iterations were used for the estimation of posterior odds) with a resulting total number of 100,000 iterations, and a prior odds ratio of 10 (prior belief that a selection model is 1/10 as likely as a neutral model for a given SNP). We considered two dairy (Churra and Latxa) and non-dairy (remaining breeds) groups.

#### Identification of selective sweeps with HapFLK and FLK

As a complementary approach, we used the hapFLK and FLK statistics to detect selective sweeps^47^,^48^. The FLK metric tests the neutrality of polymorphic markers by contrasting their allele frequencies in a set of populations against what would be expected under a neutral evolution scenario. A neigbor joining tree based on a matrix of Reynolds genetic distances is built and, under the null hypothesis of no-selection, branch length is expected to be proportional to the amount of genetic drift in each population. The hapFLK test is similar, but extends the FLK test to account for the haplotype structure in the sample. Importantly, this method is particularly robust to the effects of bottlenecks and migration and it can work with unphased data, as in the current case^47^.

To estimate hierarchical population structure, we calculated Reynolds distances and converted them to a kinship matrix with R scripts provided in the hapFLK webpage (https://forge-dga.jouy.inra.fr/projects/hapflk). In the hapFLK analysis, the number of haplotype clusters was set to 20 using the cross-validation procedure assumed in the fastPHASE model^19^ and the hapFLK statistic was calculated as the average of 30 expectation maximization iterations. The calculation of raw P-values was based on the null distribution of empirical values^47^. We made sure that these *P*-values were uniformly distributed by plotting them in a histogram (Supplementary Fig. S4). Multiple testing correction was done by using a false discovery rate approach^49^. The obtained values were plotted with the aid of an R script. Neighbor-joining trees were built by using matrices of pairwise Reynolds distances based on either the full SNP dataset (genome tree) or those SNPs mapping to putative selective sweeps (local trees). A detailed description about how local population trees are built can be found at the following website: https://forge-dga.jouy.inra.fr/projects/hapflk/wiki/LocalTrees.

#### Statistical analysis of overlaps between selective sweeps detected in the current work and those identified in previous studies

In order to assess if the amount of overlaps between the selective sweeps detected by us and those reported in previous studies^7^,^8^ was higher than what would be attributable to chance, a circular permutation approach was implemented^50^. This re-sampling procedure assumes the following steps:

1. The genome is considered to be circular and it is ordered chromosome-by-chromosome; additionally the selective sweeps previously identified by other authors^7^,^8^ are located (**set 1**).
2. A random value “d” between 1 and the maximum number of SNPs is chosen and all selective sweeps identified by us (**set 2**) are shifted to a distance equal to “d”.
3. The number of overlaps between **set 1** and **set 2** is recalculated.
4. These two steps are repeated 10,000 times with a different, randomly chosen “d” value each time, and the number of permutations in which the number of overlaps exceeds the real number of overlaps is counted.
5. Once finished, the bootstrapped distribution of the number of overlaps allows calculating a bootstrapped *P*-value for the observed number of overlaps under the null (the observed number of overlaps is not larger than that expected by chance) and the alternative (the observed number of overlaps is larger than that expected by chance) hypotheses.

## Additional Information

**How to cite this article**: Manunza, A. *et al.* Population structure of eleven Spanish ovine breeds and detection of selective sweeps with BayeScan and hapFLK. *Sci. Rep.* **6**, 27296; doi: 10.1038/srep27296 (2016).

## Supplementary Material

Supplementary Information

## Figures and Tables

**Table 1:** Outlier SNPs found with the F_ST_-based method implemented in BayeScan.

Chr	SNP	Position (Mb)	α-value	q-value	N SNPs	Gutiérrez-Gil*et al*.^8^	Fariello *et al*.^7^
1	s28145.1	256.60	1.32	0.033	3	–	–
	OAR1_77069506.1	72.00	1.28	0.038		–	–
	s61441.1	27.80	1.24	0.043		–	–
2	OAR2_194667510.1	183.60	1.49	0.006	8	–	–
	s38806.1	247.00	1.73	0.010		–	–
	OAR2_25448426.1	25.00	1.44	0.017		–	–
	OAR2_150515619.1	141.50	1.36	0.020		–	–
	s58048.1	52.40	1.39	0.021		52.3–52.5 Mb	51.4–53.4 Mb
	s17630.1	6.70	1.31	0.028		–	–
	OAR2_56768579.1	52.90	1.29	0.031		52.3–52.5 Mb	51.4–53.4 Mb
	OAR2_83850165.1	78.60	1.29	0.036		–	–
3	OAR3_203907310.1	189.30	1.47	0.007	5	–	–
	s29466.1	18.40	1.52	0.005		18.9–19.3 Mb	
	OAR3_164170826.1	153.50	1.36	0.023		153.4–154.5 Mb	151.4–156.9 Mb
	s38388.1	201.40	1.30	0.034		–	–
	s19983.1	93.80	1.26	0.041		–	–
6	OAR6_44123475_X.1	39.40	1.28	0.040	1	39.3–39.5 Mb	35.9–38.3Mb
7	OAR7_30772408.1	27.00	1.40	0.018	4		–
	s68972.1	43.70	1.63	0.001		–	–
	s11241.1	27.00	1.45	0.011		–	–
	OAR7_1827930.1	2.10	1.28	0.047		–	–
9	OAR9_14653377.1	14.30	1.40	0.014	1	–	–
10	OAR10_90168545.1	82.50	1.81	0.003	3	–	–
	OAR10_23129120.1	23.50	1.43	0.012		–	24.02–34.91
	OAR10_79676247.1	72.70	1.35	0.025		–	–
12	s34065.1	37.90	1.25	0.045	–	–	
13	s56762.1	2.00	1.52	0.004	4	–	–
	s38696.1	74.30	1.50	0.008		–	–
	s05603.1	72.00	1.37	0.015		–	–
	s19740.1	22.90	1.27	0.050		–	–
14	OAR14_9498278.1	9.10	2.04	0.000	1	–	6.3–13.6
15	s45350.1	20.60	1.23	0.048	1	16.6–20.6 Mb	
17	OAR17_33487124.1	30.60	1.44	0.010	2	–	–
	OAR17_23200636.1	20.70	1.47	0.009		–	–
19	s18836.1	51.30	1.37	0.026	2	–	–
	OAR19_33355170.1	31.60	1.50	0.006		–	30.4–35.09
24	s06827.1	13.10	1.46	0.008	1	–	–
25	s67158.1	7.70	1.43	0.013	2	–	–
	OAR25_23589759.1	22.60	1.31	0.029		–	–

In the two columns at the right part of the table, we show evidence of positional concordance with selective sweeps detected with an F_ST_-outlier approach and other methods^8^ as well as with the FLK and hapFLK metrics^7^. CHR = chromosome, N SNPs = Number of outlier SNPs.

**Table 2:** Putative selective sweeps identified in the hapFLK-based analysis.

CHR	Reg (Mb)	Flanking SNPs of SNPs	Number	Raw P-value	q-value	Gutiérrez-Gil *et al*.^8^	Fariello *et al.*^7^
2	82.8–87.7	OAR2_88062818.1–s35257.1	97	0.034	0.574	83.1–85.3 Mb	81.2–87.3 Mb
**3**	**150.5**–**154.2**	**s26286.1**–**OAR3_165050963.1**	**68**	**0.029**	**0.545**	153.4–154.5 Mb	151.4–156.9 Mb
5	46.5–49.1	s59995.1–OAR5_53435489.1	48	0.007	0.213	–	47.3–49.3 Mb
**6**	**4.3**–**49.9**	**OAR6_6402059.1**– **OAR6_55087517_X.1**	**860**	**0.000**	**0.000**	39.3–39.5 Mb	35.9–38.3 Mb
	52.3–52.6	OAR6_57796972.1–OAR6_58069886.1	7	0.049	0.604	–	–
	64.5–74.3	OAR6_70844973.1–OAR6_81183719.1	180	0.016	0.389	69.9–70.5 Mb	67.9–70.3 Mb
	112.1–115.6	OAR6_127397796.1–s33220.1	58	0.026	0.52	–	–
10	29.1–29.3	OAR10_29159858.1–OAR10_29381795.1	7	0.044	0.598	–	29.4–29.7 Mb
**13**	43.6–50.9	s39429.1–OAR13_55448085.1	104	0.006	0.192	48.9–52.0 Mb	43.3–51.2
	**68.8**–**74.9**	**OAR13_74074760.1**–**OAR13_80614774_X.1**	**97**	**0.025**	**0.524**	–	–
16	24.7–25.2	s59907.1–OAR16_27501072.1	13	0.043	0.598	–	–
17	61.2–67.1	s25636.1–s73670.1	34	0.039	0.591	–	–
24	6.9–11.0	OAR24_8063846.1–s18520.1	44	0.042	0.598	–	–
25	24.8–45.3	OAR25_25923466.1–OAR25_48288071_X.1	353	0.008	0.249	–	–
26	0.16–3.8	OAR26_222715_X.1–s54858.1	47	0.030	0.547	–	–

Those sweeps consistently found with BayeScan are shown in bold.

In the two columns at the right part of the table, we show evidence of positional concordance with previously reported selective sweeps^7^,^8^. CHR = chromosome.
