# Supplementary material for: Population structure of eleven Spanish ovine breeds and detection of selective sweeps with BayeScan and hapFLK
Source: Sci Rep. 2016 Jun 7;6:27296. doi: 10.1038/srep27296 (PMC4895181; doi:10.1038/srep27296)

**Population structure of eleven Spanish ovine breeds and detection of selective sweeps with BayeScan and hapFLK**

A. Manunza^1^,*, T.F. Cardoso^1^,*, A. Noce^1^, A. Martínez^2^, A. Pons^3^, L.A. Bermejo^4^, V. Landi^2^, A. Sànchez^1,5^, J. Jordana^5^, J.V. Delgado^2^, S. Adán^6^, J. Capote^7^, O. Vidal^8^, E. Ugarte^9^, J. J. Arranz^10^, J. H. Calvo^11^, J. Casellas^5^ and M. Amills^1,5^

^1^Department of Animal Genetics, Center for Research in Agricultural Genomics (CSIC-IRTA-UAB-UB), Campus Universitat Autònoma de Barcelona, Bellaterra 08193, Spain; ^2^Departamento de Genética, Universidad de Córdoba, Córdoba 14071, Spain; ^3^Unitat de Races Autòctones, Servei de Millora Agrària i Pesquera (SEMILLA), Son Ferriol 07198, Spain; ^4^Departamento de Ingeniería, Producción y Economía Agrarias, Universidad de La Laguna, 38071 La Laguna, Tenerife, Spain; ^5^Departament de Ciència Animal i dels Aliments, Universitat Autònoma de Barcelona, Bellaterra 08193, Spain; ^6^Federación de Razas Autóctonas de Galicia (BOAGA), Pazo de Fontefiz, 32152 Coles. Ourense, Spain; ^7^Instituto Canario de Investigaciones Agrarias, La Laguna 38108, Tenerife, Spain; ^8^Departament de Biologia, Universitat de Girona, Girona 17071, Spain; ^9^Neiker-Tecnalia, Campus Agroalimentario de Arkaute, apdo 46 E-01080 Vitoria-Gazteiz (Araba), Spain; ^9^Departamento de Producción Animal, Universidad de León, León 24071, Spain; Centro de Investigación y Tecnología Agroalimentaria de Aragón (CITA), Unidad de Tecnología en Producción Animal, Avda. Montañana, 930, 50059 Zaragoza, Spain.

Supplementary Table S1. Putative selective sweeps identified in the FLK-based analysis

| **CHR** | **Reg (Mb)** | **Flanking SNPs** | **Number**  **of SNPs** | **Raw P-value** | **q-value** |
| --- | --- | --- | --- | --- | --- |
| 1 | 6.6 - 8.8 | s22471.1 - s61237.1 | 3 | 1.08E-08 | 1.38E-04 |
|  | 13.5 - 18.5 | OAR1_13393044.1 - s14459.1 | 3 | 7.90E-07 | 7.98E-03 |
|  | 22.7 | s27454.1 | 1 | 2.02E-06 | 1.63E-02 |
|  | 26.9 - 34.4 | OAR1_27215234.1 - OAR1_35419260.1 | 7 | 6.83E-09 | 7.85E-05 |
|  | 39.9 - 43.4 | OAR1_41220308.1 - OAR1_45043922.1 | 5 | 8.58E-08 | 1.13E-03 |
|  | 58.2 - 63.8 | OAR1_61493280.1 - s27673.1 | 4 | 2.73E-08 | 3.82E-04 |
|  | 67.4 - 69.9 | OAR1_71967991.1 - OAR1_74545077.1 | 3 | 2.14E-08 | 2.88E-04 |
|  | 73.5 - 81.1 | s68272.1 - OAR1_86417977.1 | 6 | 4.67E-09 | 4.80E-05 |
|  | 87.0 - 87.2 | s08905.1 - OAR1_92567044.1 | 2 | 1.08E-06 | 1.01E-02 |
|  | 93.5 | OAR1_99324339.1 | 1 | 5.62E-06 | 3.38E-02 |
|  | 100.2 - 100.2 | OAR1_107574976.1 - OAR1_107588571.1 | 2 | 8.00E-08 | 1.05E-03 |
|  | 104.8 | s55826.1 | 1 | 7.59E-06 | 4.01E-02 |
|  | 116.1 | OAR1_125170115.1 | 1 | 6.91E-08 | 9.53E-04 |
|  | 123.0 - 135.1 | OAR1_133238194.1 - s16899.1 | 8 | 2.48E-07 | 3.04E-03 |
|  | 142.5 - 145.7 | OAR1_153903725.1 -OAR1_157286052.1 | 3 | 2.02E-06 | 1.63E-02 |
|  | 155.1 - 156.5 | OAR1_167129505.1 - OAR1_168642518.1 | 2 | 7.29E-06 | 3.90E-02 |
|  | 160.7 - 161.1 | s32338.1 - OAR1_174516204.1 | 2 | 4.35E-06 | 2.81E-02 |
|  | 169.9 | OAR1_183201498.1 | 1 | 3.88E-10 | 2.08E-06 |
|  | 180.6 - 182.6 | s22135.1 - s57575.1 | 2 | 1.35E-09 | 9.84E-06 |
|  | 187.4 | s00505.1 | 1 | 4.15E-06 | 2.72E-02 |
|  | 191.8 - 194.8 | OAR1_207103971.1 - OAR1_210312977.1 | 4 | 1.30E-12 | 2.60E-10 |
|  | 199.9 - 200.8 | OAR1_215776072.1 - s62524.1 | 2 | 6.23E-11 | 1.55E-07 |
|  | 207.9 | OAR1_224530290.1 | 1 | 1.03E-06 | 9.84E-03 |
|  | 210.1 - 213.2 | OAR1_226804688.1 -OAR1_229955101.1 | 3 | 3.34E-07 | 3.99E-03 |
|  | 218.1 - 219.3 | OAR1_235302813.1 - OAR1_236540864.1 | 2 | 2.77E-06 | 2.07E-02 |
|  | 225.4 - 225.7 | s23022.1 - OAR1_243245548.1 | 2 | 2.05E-06 | 1.65E-02 |
|  | 229.2 - 229.2 | OAR1_247165341.1 - OAR1_247181814.1 | 2 | 1.95E-06 | 1.59E-02 |
|  | 240.6 - 255.3 | OAR1_260245778.1- OAR1_275970264.1 | 11 | 3.42E-10 | 1.81E-06 |
|  | 260.2 - 264.9 | s55111.1 - OAR1_286637130.1 | 4 | 1.30E-09 | 9.57E-06 |
| 2 | 3.4 - 7.0 | s04138.1 - OAR2_5857776.1 | 4 | 3.60E-09 | 3.45E-05 |
|  | 10-1 - 16.8 | OAR2_9214027_X.1 - s14482.1 | 6 | 3.89E-08 | 5.48E-04 |
|  | 23.6 - 26.9 | OAR2_24075355.1 - OAR2_27877354.1 | 2 | 2.85E-06 | 2.12E-02 |
|  | 32.9 - 38.2 | OAR2_34260595.1 - OAR2_39793505.1 | 6 | 3.27E-09 | 3.14E-05 |
|  | 44.4 - 49.0 | s00051.1 - s18764.1 | 4 | 5.30E-08 | 7.35E-04 |
|  | 51.6 - 64.4 | OAR2_55248792.1 -OAR2_69954696_X.1 | 14 | 3.47E-08 | 4.82E-04 |
|  | 72.6 | s39140.1 | 1 | 5.21E-08 | 7.23E-04 |
|  | 80.0 - 84.5 | OAR2_85242915.1 - OAR2_89889867.1 | 9 | 1.75E-12 | 3.65E-10 |
|  | 94.7 - 125.9 | OAR2_101907081.1 - OAR2_134185168.1 | 24 | 1.25E-08 | 1.63E-04 |
|  | 132.5 | OAR2_141031378.1 | 1 | 6.03E-06 | 3.53E-02 |
|  | 140.0 - 142.0 | OAR2_148853991.1 - OAR2_151022859.1 | 3 | 2.51E-07 | 3.07E-03 |
|  | 146.3 - 149.0 | OAR2_155371114.1 - OAR2_158051810.1 | 3 | 2.53E-06 | 1.95E-02 |
|  | 155.8 - 167.9 | OAR2_165244995.1 - OAR2_177722937.1 | 9 | 3.25E-09 | 3.14E-05 |
|  | 176.6 - 176.6 | s00137.1 - OAR2_187375823.1 | 2 | 7.36E-08 | 9.96E-04 |
|  | 181.1 | OAR2_191985251.1 | 1 | 6.71E-06 | 3.74E-02 |
|  | 185.5 | s06126.1 | 1 | 3.62E-06 | 2.49E-02 |
|  | 190.1 - 190.2 | OAR2_201460959.1 - OAR2_201691596.1 | 2 | 6.18E-07 | 6.52E-03 |
|  | 198.1 - 209.9 | OAR2_209681146.1 - OAR2_222238051.1 | 7 | 8.40E-08 | 1.11E-03 |
|  | 214.0 - 214.2 | OAR2_226528973.1 - s38960.1 | 2 | 6.25E-06 | 3.60E-02 |
|  | 217.9 | s34413.1 | 1 | 4.40E-07 | 4.94E-03 |
|  | 221.4 | OAR2_234056829.1 | 1 | 1.15E-08 | 1.49E-04 |
|  | 227.2 - 229.4 | OAR2_239994767.1 - OAR2_242236604.1 | 2 | 2.68E-06 | 2.03E-02 |
|  | 233.7 | s03354.1 | 1 | 1.05E-06 | 9.94E-03 |
|  | 238.8 - 248.2 | s05610.1 - s58576.1 | 13 | 8.69E-09 | 1.07E-04 |
| 3 | 0.06 | s68356.1 | 1 | 8.35E-07 | 8.28E-03 |
|  | 0.8 | s64335.1 | 1 | 1.00E-10 | 3.10E-07 |
|  | 10.7 | OAR3_11261007.1 | 1 | 1.60E-06 | 1.35E-02 |
|  | 20.8 - 28.4 | OAR3_22495716.1- OAR3_30631890.1 | 6 | 3.37E-08 | 4.75E-04 |
|  | 32.2 - 37.3 | s69167.1 - s22319.1 | 4 | 2.98E-10 | 1.55E-06 |
|  | 56.5 - 58.4 | OAR3_59844188.1 - s63690.1 | 2 | 5.77E-12 | 3.30E-09 |
|  | 65.2 - 66.0 | s28628.1 - OAR3_69758613_X.1 | 3 | 6.75E-08 | 9.51E-04 |
|  | 75.2 - 76.3 | OAR3_79418497.1 - s73802.1 | 2 | 1.34E-09 | 9.84E-06 |
|  | 81.8 | OAR3_86556183.1 | 1 | 1.08E-05 | 4.96E-02 |
|  | 87.8 - 92.1 | OAR3_93074872.1 - OAR3_97845168.1 | 3 | 3.35E-06 | 2.35E-02 |
|  | 96.9 | OAR3_103153461.1 | 1 | 9.65E-06 | 4.65E-02 |
|  | 103.5-110.8 | s32880.1 - OAR3_118084612.1 | 4 | 8.00E-08 | 1.05E-03 |
|  | 115.6 - 119.5 | OAR3_123322994.1 - OAR3_127397300.1 | 4 | 3.16E-07 | 3.78E-03 |
|  | 124.6 - 126.4 | OAR3_132833292.1 - OAR3_134910604.1 | 3 | 3.76E-11 | 7.16E-08 |
|  | 132.1 - 154.2 | OAR3_141240034.1 - OAR3_165009241.1 | 16 | 7.43E-10 | 4.67E-06 |
|  | 159.2 - 160.9 | OAR3_170381258.1 - OAR3_172159515_X.1 | 4 | 3.94E-08 | 5.54E-04 |
|  | 168.5 | OAR3_181081760.1 | 1 | 8.78E-06 | 4.39E-02 |
|  | 174.0 - 182.3 | OAR3_186733102.1 - OAR3_196341344.1 | 8 | 1.95E-13 | 7.06E-12 |
|  | 186.5 - 192.1 | OAR3_200790828.1 - OAR3_206856083.1 | 5 | 3.78E-11 | 7.16E-08 |
|  | 196.0 - 197.7 | OAR3_211069150.1 - OAR3_212887201_X.1 | 2 | 1.20E-06 | 1.10E-02 |
|  | 201.4 - 201.5 | OAR3_216714207.1 - OAR3_216958700.1 | 2 | 4.03E-06 | 2.68E-02 |
|  | 212.1 - 216.7 | OAR3_229873996.1 - s15228.1 | 4 | 4.09E-07 | 4.63E-03 |
|  | 220.3 - 222.1 | OAR3_239536012.1 - DU366895_507.1 | 2 | 7.54E-08 | 1.01E-03 |
| 4 | 0.7 | OAR4_693681.1 | 1 | 7.59E-06 | 4.01E-02 |
|  | 4.9 - 7.1 | s51727.1 - OAR4_6864529.1 | 3 | 3.42E-08 | 4.79E-04 |
|  | 12.9 - 15.3 | OAR4_13174894.1 -s51401.1 | 3 | 6.02E-08 | 8.52E-04 |
|  | 20.2 - 20.5 | OAR4_21038955.1 - OAR4_21394967.1 | 2 | 1.54E-08 | 2.00E-04 |
|  | 24.0 - 35.5 | s57130.1 - OAR4_37516230.1 | 7 | 3.06E-10 | 1.58E-06 |
|  | 56.2 - 58.9 | s66683.1 - OAR4_62280257.1 | 2 | 1.89E-06 | 1.55E-02 |
|  | 63.0 | OAR4_66673320.1 | 1 | 3.56E-06 | 2.46E-02 |
|  | 69.6 - 76.2 | s56593.1 - OAR4_80854660.1 | 6 | 3.55E-08 | 4.95E-04 |
|  | 8.3 - 81.4 | OAR4_86339728.1 - s31519.1 | 3 | 1.32E-06 | 1.17E-02 |
|  | 86.3 - 87.4 | OAR4_91739253.1 - OAR4_92869288.1 | 2 | 4.07E-07 | 4.63E-03 |
|  | 92.3 - 96.3 | OAR4_97984717.1 - s72158.1 | 4 | 3.14E-09 | 3.02E-05 |
|  | 101.2 -103.5 | s14348.1 - s58949.1 | 2 | 1.02E-06 | 9.75E-03 |
|  | 111.4 - 119.1 | s30066.1 - OAR4_127053991.1 | 9 | 1.21E-07 | 1.60E-03 |
| 5 | 5.0 | s08913.1 | 1 | 7.11E-06 | 3.85E-02 |
|  | 10.0 - 10.1 | OAR5_11546988.1 - OAR5_11655482.1 | 2 | 3.05E-06 | 2.21E-02 |
|  | 14.0 - 16.5 | s75256.1 - OAR5_19010560.1 | 4 | 1.01E-06 | 9.67E-03 |
|  | 21.8 - 37.3 | s67491.1 - OAR5_41108751.1 | 10 | 1.90E-08 | 2.52E-04 |
|  | 43.4 - 49.2 | s17197.1 - OAR5_53543443.1 | 13 | 1.03E-07 | 1.37E-03 |
|  | 60.5 | s57895.1 | 1 | 3.08E-06 | 2.23E-02 |
|  | 67.8 - 69.1 | s12711.1 - s32867.1 | 4 | 2.12E-07 | 2.67E-03 |
|  | 79.6 - 80.4 | OAR5_87470215.1 - OAR5_88375559_X.1 | 3 | 2.73E-11 | 4.58E-08 |
|  | 86.0 | OAR5_94270780.1 | 1 | 4.04E-07 | 4.63E-03 |
|  | 90.0 | OAR5_98200116.1 | 1 | 2.10E-06 | 1.68E-02 |
|  | 98.4 - 102.9 | OAR5_107273510.1 - s58645.1 | 4 | 1.93E-07 | 2.42E-03 |
|  | 106.8 | OAR5_116287948_X.1 | 1 | 1.84E-08 | 2.49E-04 |
| 6 | 0.8 - 11-3 | OAR6_568937.1 - OAR6_13831648.1 | 8 | 2.91E-08 | 4.00E-04 |
|  | 16.0 - 18.5 | OAR6_18935576.1 - OAR6_21531444.1 | 4 | 1.84E-10 | 8.24E-07 |
|  | 23.5 | s22136.1 | 1 | 1.95E-08 | 2.58E-04 |
|  | 29.0 - 75.7 | OAR6_32862854.1 - OAR6_82677771.1 | 51 | 1.45E-14 | 0.00E+00 |
|  | 79.6 - 81.2 | OAR6_86998248.1 - OAR6_88678679.1 | 5 | 3.30E-09 | 3.14E-05 |
|  | 87.4 - 89.2 | OAR6_95813186.1 - OAR6_97862827.1 | 3 | 3.24E-06 | 2.30E-02 |
|  | 103.5 - 111.0 | OAR6_114023927.1 - OAR6_126093150.1 | 5 | 4.60E-09 | 4.74E-05 |
| 7 | 6 - 12.9 | OAR7_6314338.1 - s61696.1 | 5 | 4.48E-07 | 5.00E-03 |
|  | 17.7 - 20.5 | OAR7_18423084.1 - s42488.1 | 2 | 4.06E-08 | 5.68E-04 |
|  | 25.3 - 29.7 | OAR7_29078940.1 - OAR7_33553470.1 | 5 | 9.92E-08 | 1.33E-03 |
|  | 33.3 - 34.2 | OAR7_37544340.1 - OAR7_38412136.1 | 2 | 4.71E-07 | 5.14E-03 |
|  | 39.7 | OAR7_43964022.1 | 1 | 1.19E-06 | 1.10E-02 |
|  | 44.2- 47.7 | s18948.1 - OAR7_52759167.1 | 3 | 7.21E-08 | 9.92E-04 |
|  | 55.5 - 58.4 | OAR7_61499056.1 - OAR7_64371135.1 | 3 | 8.58E-06 | 4.34E-02 |
|  | 62.4 | OAR7_68342073.1 | 1 | 1.05E-06 | 9.94E-03 |
|  | 67.6 | OAR7_74151838.1 | 1 | 8.45E-06 | 4.30E-02 |
|  | 73.3 | OAR7_80225457.1 | 1 | 7.57E-06 | 4.01E-02 |
|  | 85.0 - 92.3 | s46801.1 - OAR7_100552217_X.1 | 6 | 2.73E-11 | 4.58E-08 |
|  | 96.0 - 99.2 | OAR7_104633749.1 - s74502.1 | 3 | 4.72E-10 | 2.68E-06 |
| 8 | 3.2 - 3.8 | OAR8_3577608.1 - OAR8_4090191.1 | 2 | 1.27E-06 | 1.14E-02 |
|  | 9.6 | OAR8_10787192.1 | 1 | 2.74E-07 | 3.32E-03 |
|  | 13.8 - 24.1 | OAR8_15422666.1 - OAR8_26651354.1 | 15 | 1.49E-08 | 1.96E-04 |
|  | 28.7 - 31.4 | OAR8_31201923.1 - OAR8_34202419.1 | 3 | 1.73E-07 | 2.18E-03 |
|  | 35.4 | OAR8_38136082_X.1 | 1 | 7.86E-07 | 7.98E-03 |
|  | 41.0 | OAR8_44173851.1 | 1 | 2.26E-06 | 1.78E-02 |
|  | 49.2 - 57.9 | OAR8_52791160.1 - OAR8_62240378.1 | 7 | 1.17E-07 | 1.56E-03 |
|  | 61.2 - 63.1 | OAR8_66018085.1 - s28127.1 | 2 | 1.39E-09 | 1.02E-05 |
|  | 67.0 - 71.1 | s59834.1 - OAR8_76325701.1 | 6 | 2.26E-09 | 2.01E-05 |
|  | 75.4 - 76.2 | s06254.1 - OAR8_82357525_X.1 | 2 | 7.42E-10 | 4.67E-06 |
|  | 80.0 - 85.5 | s62947.1 - OAR8_92283226.1 | 5 | 1.19E-09 | 8.53E-06 |
| 9 | 2.2 | OAR9_2098219.1 | 1 | 5.66E-07 | 6.04E-03 |
|  | 10.4 | OAR9_10458861.1 | 1 | 9.49E-07 | 9.25E-03 |
|  | 15.9 - 18.1 | s22485.1 - OAR9_18982135.1 | 2 | 9.30E-10 | 6.04E-06 |
|  | 34.3 - 37.8 | OAR9_36110320.1 - OAR9_39790883.1 | 3 | 1.21E-07 | 1.60E-03 |
|  | 46.6 | OAR9_49061755.1 | 1 | 8.77E-06 | 4.39E-02 |
|  | 50.7 - 61.0 | s31696.1 - OAR9_64150094.1 | 8 | 4.64E-07 | 5.09E-03 |
|  | 66.6 - 77.6 | OAR9_70612779.1 - OAR9_82282148_X.1 | 7 | 9.78E-08 | 1.31E-03 |
|  | 83.5 | OAR9_88387736.1 | 1 | 2.33E-06 | 1.83E-02 |
|  | 88.8 | s70574.1 | 1 | 2.32E-06 | 1.82E-02 |
| 10 | 2.9 - 8.8 | s38460.1 - OAR10_7112669.1 | 4 | 6.83E-08 | 9.51E-04 |
|  | 14.0 | OAR10_12595183.1 | 1 | 0 | 0.00E+00 |
|  | 19.6 - 20.4 | OAR10_18969912.1 - OAR10_19765032.1 | 3 | 7.90E-14 | 1.41E-12 |
|  | 24.2 - 32.1 | OAR10_24249580_X.1 - OAR10_32357172.1 | 17 | 9.32E-13 | 1.63E-10 |
|  | 40.3 - 42.7 | OAR10_41174634.1 - OAR10_43601954.1 | 2 | 4.18E-08 | 5.79E-04 |
|  | 48.1 - 77.7 | OAR10_48888461.1 - OAR10_84982826.1 | 21 | 2.14E-08 | 2.88E-04 |
|  | 81.7 - 85.6 | OAR10_89336296.1 - s40732.1 | 5 | 1.40E-07 | 1.82E-03 |
| 11 | 4.7 | OAR11_4099848.1 | 1 | 3.79E-07 | 4.38E-03 |
|  | 8.8 - 9.4 | s13587.1 - OAR11_9050522.1 | 2 | 1.54E-06 | 1.32E-02 |
|  | 16.3 - 23.9 | s13840.1 - s21117.1 | 10 | 1.11E-15 | 0.00E+00 |
|  | 29.5 - 30.1 | s62452.1 - OAR11_32009069.1 | 2 | 3.43E-07 | 4.10E-03 |
|  | 36.1 | OAR11_38622874.1 | 1 | 7.74E-06 | 4.05E-02 |
|  | 40.4 - 42.9 | OAR11_42915917.1 - s46121.1 | 3 | 3.11E-10 | 1.59E-06 |
|  | 49.1 | OAR11_52263542.1 | 1 | 1.91E-07 | 2.39E-03 |
|  | 56.8 | s75385.1 | 1 | 8.27E-06 | 4.25E-02 |
| 12 | 7.0 - 19.8 | OAR12_9064662_X.1 - OAR12_22906100.1 | 9 | 2.82E-08 | 3.89E-04 |
|  | 24.6 | OAR12_28120988.1 | 1 | 5.35E-06 | 3.26E-02 |
|  | 28.0 | s67736.1 | 1 | 1.44E-06 | 1.24E-02 |
|  | 34.4 - 34.8 | s66941.1 - OAR12_39002985.1 | 2 | 2.59E-08 | 3.65E-04 |
|  | 38.3 - 47.9 | s07697.1 - s06305.1 | 7 | 2.99E-08 | 4.12E-04 |
|  | 51.3 | s49309.1 | 1 | 1.08E-05 | 4.98E-02 |
|  | 57.0 - 57.1 | OAR12_63472054.1 - s25216.1 | 2 | 2.49E-06 | 1.92E-02 |
|  | 63.8 - 75.5 | OAR12_70366176.1 - OAR12_83198888.1 | 11 | 4.76E-11 | 1.04E-07 |
| 13 | 1.0 - 6.6 | OAR13_1535133.1 - s59225.1 | 6 | 1.37E-07 | 1.81E-03 |
|  | 12.6 - 24.0 | OAR13_16254632.1 - OAR13_26553208.1 | 9 | 3.07E-09 | 2.95E-05 |
|  | 30.3 - 34.6 | OAR13_33557008.1 - OAR13_38060342.1 | 5 | 3.81E-08 | 5.36E-04 |
|  | 38.4 | s37104.1 | 1 | 8.23E-07 | 8.16E-03 |
|  | 44.9 - 49.5 | OAR13_48288558.1 - OAR13_52975823.1 | 13 | 8.42E-10 | 5.36E-06 |
|  | 54.6 - 56.4 | s50410.1 - s14721.1 | 4 | 9.27E-07 | 9.05E-03 |
|  | 62.3 - 75.8 | s28243.1 - s08015.1 | 11 | 2.06E-09 | 1.77E-05 |
| 14 | 4.1 - 5.1 | OAR14_4053146.1 - s59715.1 | 2 | 5.83E-07 | 6.19E-03 |
|  | 9.5 | OAR14_9811909.1 | 1 | 3.33E-07 | 3.99E-03 |
|  | 14.6 | OAR14_14862862.1 | 1 | 6.49E-06 | 3.69E-02 |
|  | 19.4 - 23.0 | OAR14_19829145.1 - OAR14_23628696.1 | 4 | 7.59E-10 | 4.76E-06 |
|  | 32.8 | DU232315_442.1 | 1 | 2.92E-06 | 2.16E-02 |
|  | 36.8 - 41.2 | OAR14_38256049.1 - s17562.1 | 3 | 5.01E-07 | 5.42E-03 |
|  | 46.2 | OAR14_48562410.1 | 1 | 1.88E-08 | 2.51E-04 |
|  | 52.6 - 55-1 | s21032.1 - s69091.1 | 4 | 1.09E-08 | 1.39E-04 |
|  | 61.0 | OAR14_67561824.1 | 1 | 3.39E-09 | 3.22E-05 |
| 15 | 2.9 | OAR15_2305340.1 | 1 | 7.22E-06 | 3.89E-02 |
|  | 7.2 - 8.6 | OAR15_6814257.1 - OAR15_8403885.1 | 2 | 4.36E-07 | 4.90E-03 |
|  | 12.6 | s33365.1 | 1 | 5.18E-06 | 3.20E-02 |
|  | 23.7 | OAR15_24866718.1 | 1 | 6.92E-06 | 3.81E-02 |
|  | 29.8 - 30.5 | s27003.1 - OAR15_32026400.1 | 2 | 1.16E-07 | 1.54E-03 |
|  | 35.0 - 35.1 | s64675.1 - OAR15_36969378.1 | 2 | 3.88E-10 | 2.08E-06 |
|  | 41.6 - 56.4 | OAR15_43835525.1 - OAR15_61815950.1 | 7 | 2.14E-10 | 9.99E-07 |
|  | 61.3 - 66.5 | OAR15_66653722.1 - OAR15_72093475.1 | 3 | 2.05E-06 | 1.65E-02 |
|  | 72.8 - -80.0 | s27911.1 - OAR15_89417210.1 | 10 | 2.24E-06 | 1.76E-02 |
| 16 | 0.8 - 4.2 | s55737.1 - s43430.1 | 3 | 1.94E-08 | 2.58E-04 |
|  | 8.2 | OAR16_8840244.1 | 1 | 3.11E-06 | 2.24E-02 |
|  | 12.0 - 13.0 | OAR16_13101603.1 - OAR16_14121217_X.1 | 3 | 1.65E-07 | 2.10E-03 |
|  | 18.0 - 18.5 | s60618.1 - OAR16_20449381.1 | 2 | 1.90E-06 | 1.56E-02 |
|  | 23.9 - 34.1 | OAR16_26006299.1 - OAR16_37125504.1 | 6 | 7.09E-11 | 1.87E-07 |
|  | 40.3 - 43.0 | OAR16_43833978.1 - OAR16_46955783.1 | 3 | 1.01E-07 | 1.35E-03 |
|  | 48.0 - 53.7 | OAR16_52272103.1 - OAR16_58608711.1 | 4 | 1.20E-07 | 1.59E-03 |
|  | 61.7 - 65.8 | s33640.1 - s46178.1 | 2 | 6.20E-07 | 6.52E-03 |
| 17 | 4.8 | OAR17_5388531.1 | 1 | 2.73E-06 | 2.05E-02 |
|  | 14.7 - 24.1 | OAR17_16420066.1 - s75053.1 | 9 | 5.46E-10 | 3.19E-06 |
|  | 31.3 | OAR17_34248651.1 | 1 | 1.00E-05 | 4.78E-02 |
|  | 39.6 - 46.8 | OAR17_42846302.1 - s09923.1 | 4 | 4.33E-06 | 2.80E-02 |
|  | 50.0 | OAR17_54526765.1 | 1 | 3.47E-10 | 1.81E-06 |
|  | 54.9 71.6 | s53948.1 - s33026.1 | 16 | 2.33E-10 | 1.12E-06 |
| 18 | 3.1 - 5.6 | OAR18_2735778_X.1 - OAR18_5282385.1 | 3 | 4.24E-10 | 2.35E-06 |
|  | 10.1 | s71923.1 | 1 | 8.85E-08 | 1.17E-03 |
|  | 15.2 | OAR18_15289260.1 | 1 | 1.17E-06 | 1.09E-02 |
|  | 19.3 - 22.5 | s31152.1 - OAR18_24812063.1 | 3 | 1.52E-09 | 1.16E-05 |
|  | 34.6 - 35.3 | OAR18_36215348.1 - OAR18_37084174.1 | 3 | 1.35E-07 | 1.79E-03 |
|  | 40.2 - 53.8 | OAR18_42860610.1 - OAR18_57583120.1 | 11 | 1.39E-12 | 2.69E-10 |
|  | 57.8 | s19546.1 | 1 | 2.43E-07 | 2.99E-03 |
| 19 | 1.1 | OAR19_1078590.1 | 1 | 1.54E-06 | 1.32E-02 |
|  | 5.2 | s38661.1 | 1 | 8.00E-06 | 4.15E-02 |
|  | 11.7 - 13.6 | s08374.1 - s71430.1 | 3 | 1.87E-07 | 2.35E-03 |
|  | 17.2 - 21.9 | OAR19_18010247.1 - s01850.1 | 8 | 5.17E-07 | 5.55E-03 |
|  | 25.0 - 31.6 | OAR19_26525006.1 - OAR19_33355170.1 | 5 | 6.83E-12 | 4.27E-09 |
|  | 41.9 - 42.1 | OAR19_44070243.1 - OAR19_44354998.1 | 2 | 1.06E-08 | 1.37E-04 |
|  | 54.3 | s31207.1 | 1 | 2.39E-06 | 1.86E-02 |
| 20 | 0.3 - 5.4 | OAR20_192404.1 - OAR20_5451328.1 | 3 | 6.00E-11 | 1.51E-07 |
|  | 12.1 - 15.4 | s38013.1 - s31449.1 | 2 | 2.56E-07 | 3.13E-03 |
|  | 22.1 - 23.8 | OAR20_23408992.1 - OAR20_25345261.1 | 3 | 2.79E-10 | 1.43E-06 |
|  | 30.6 - 32.9 | OAR20_33616292.1 -OAR20_36218603.1 | 6 | 1.22E-06 | 1.11E-02 |
|  | 36.5 - 44.8 | OAR20_40033147.1 - OAR20_48754392.1 | 8 | 3.02E-11 | 5.24E-08 |
|  | 49.7 - 49.8 | s74404.1 - OAR20_54332894.1 | 2 | 9.34E-06 | 4.58E-02 |
| 21 | 1.8 - 3.4 | s26215.1 - OAR21_4255160.1 | 3 | 2.75E-10 | 1.43E-06 |
|  | 11.5 | OAR21_13084748.1 | 1 | 9.97E-07 | 9.67E-03 |
|  | 21.5 | OAR21_24192895.1 | 1 | 1.54E-06 | 1.32E-02 |
|  | 27.1 - 32.9 | OAR21_30414443.1 - s32618.1 | 4 | 1.41E-06 | 1.22E-02 |
|  | 41.6 - 47.2 | s28053.1 - OAR21_52385914.1 | 5 | 1.87E-08 | 2.51E-04 |
| 22 | 1.0 | s28956.1 | 1 | 4.28E-06 | 2.78E-02 |
|  | 8.1 - 9.6 | OAR22_9813363.1 - s34869.1 | 2 | 5.61E-10 | 3.27E-06 |
|  | 16.7 - 25.2 | s63432.1 - OAR22_29530029.1 | 6 | 1.30E-12 | 2.60E-10 |
|  | 30.9 | s11014.1 | 1 | 1.02E-05 | 4.83E-02 |
|  | 38.1 | s34551.1 | 1 | 5.09E-06 | 3.17E-02 |
|  | 43.4 | s26430.1 | 1 | 3.69E-06 | 2.53E-02 |
| 23 | 2.1 | OAR23_2381157.1 | 1 | 9.08E-06 | 4.49E-02 |
|  | 6.0 - 12.4 | s01824.1 - OAR23_13597788.1 | 5 | 3.16E-08 | 4.41E-04 |
|  | 27.3 - 38.8 | OAR23_28582088.1 - OAR23_41094177.1 | 8 | 8.92E-10 | 5.75E-06 |
|  | 44.7 - 47.5 | OAR23_47354853.1 - s58136.1 | 5 | 9.84E-11 | 3.09E-07 |
|  | 5.7 - 7.9 | OAR24_6671055.1 - OAR24_9134934.1 | 4 | 1.70E-09 | 1.35E-05 |
| 24 | 11.8 - 17.9 | s61066.1 - s33350.1 | 3 | 1.38E-08 | 1.79E-04 |
|  | 23.6 - 23.8 | s39918.1 - OAR24_26210500.1 | 2 | 4.63E-07 | 5.09E-03 |
|  | 38.7 | s71282.1 | 1 | 2.32E-07 | 2.89E-03 |
|  | 7.3 - 13.1 | s30024.1 - OAR25_13459673.1 | 5 | 1.84E-08 | 2.49E-04 |
| 25 | 19.0 - 42.2 | s12866.1 - OAR25_44662776.1 | 19 | 9.49E-11 | 3.01E-07 |
|  | 10.2 - 10.9 | OAR26_12757818.1 - OAR26_13494685.1 | 2 | 1.53E-08 | 2.00E-04 |
| 26 | 20.8 - 29.5 | OAR26_24584502.1 - OAR26_33808544.1 | 8 | 1.41E-08 | 1.82E-04 |
|  | 42.5 | OAR26_48248025.1 | 1 | 8.46E-06 | 4.30E-02 |
|  |  |  |  |  |  |


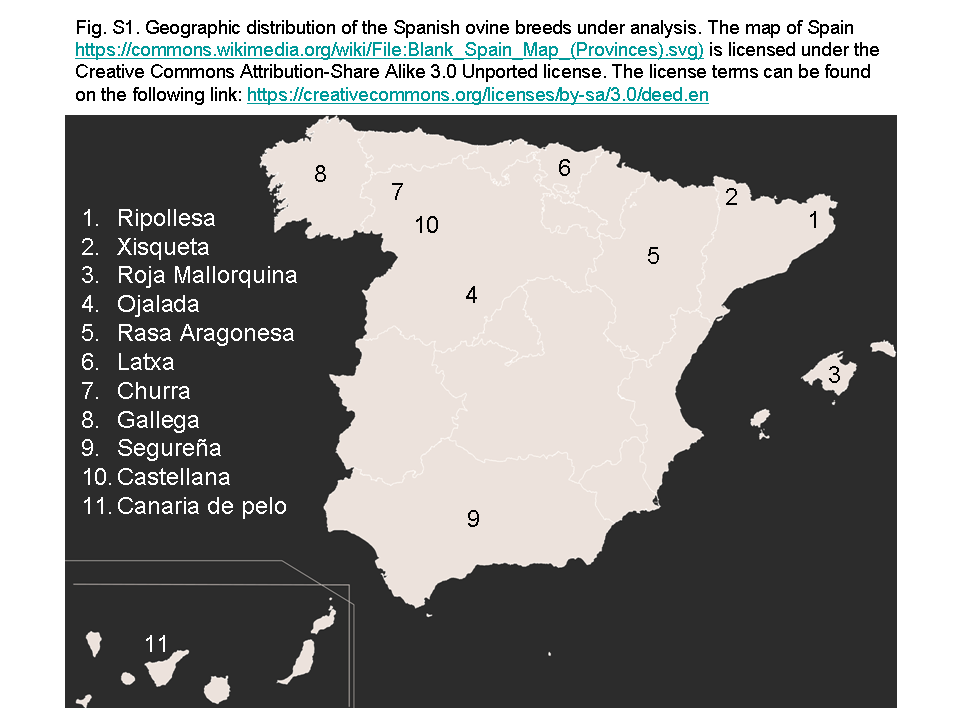


Fig. S3. Allele frequencies of the SNPs located within three selective sweeps in Oar3, Oar6 and Oar13


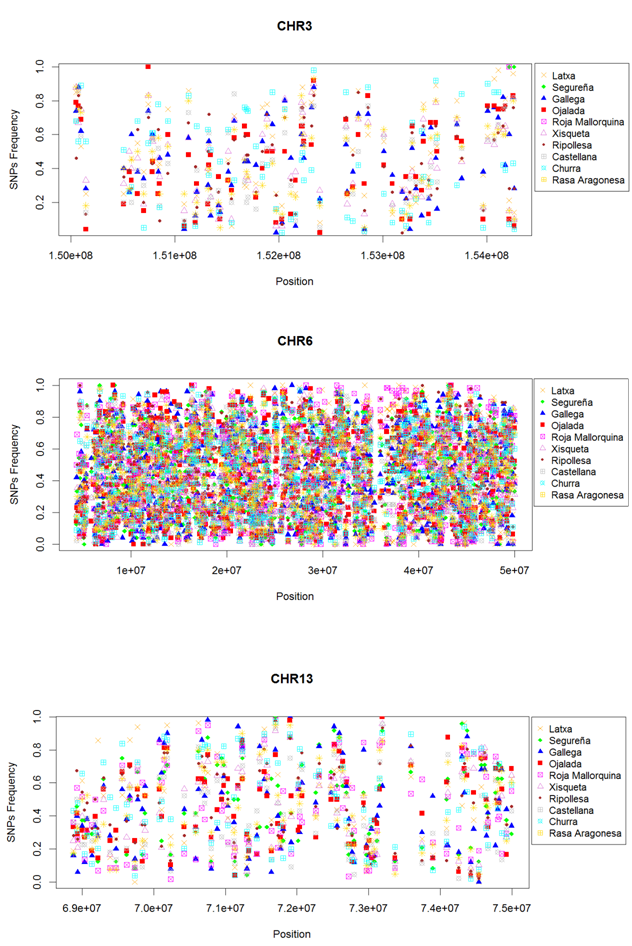


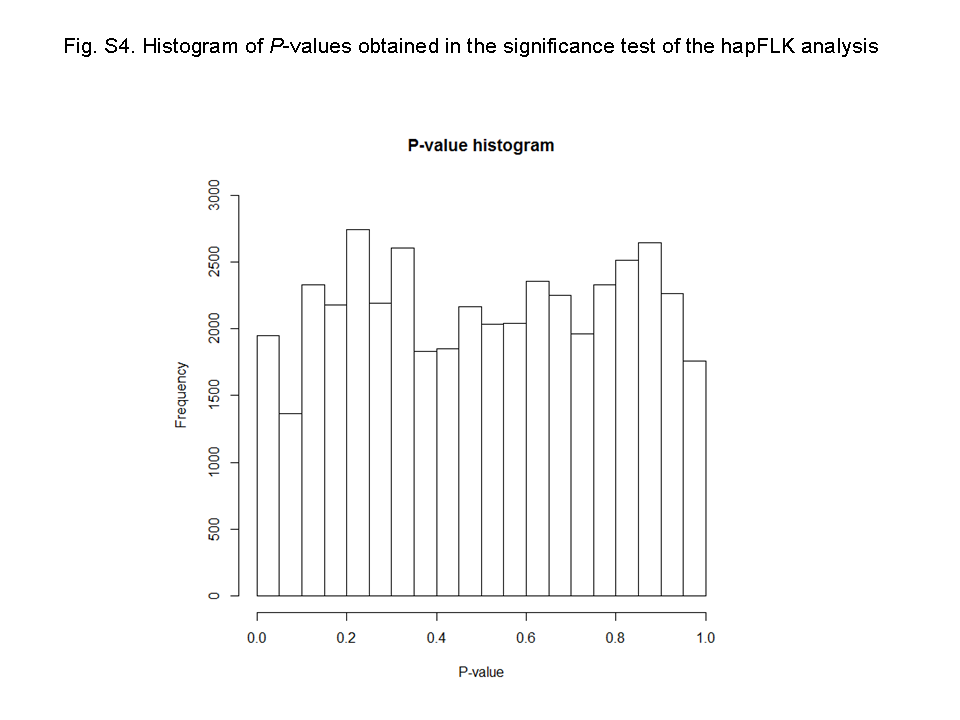

Supplement: Supplementary Information [file srep27296-s1.doc]
